# Vitreoschisis‐induced vitreous cortex remnants: missing link in proliferative vitreoretinopathy

**DOI:** 10.1111/aos.14216

**Published:** 2019-08-19

**Authors:** Koen van Overdam

**Affiliations:** ^1^ Department of Vitreoretinal Surgery The Rotterdam Eye Hospital Rotterdam The Netherlands

Editor,

Despite significant advances in vitreoretinal instrumentation, techniques and surgical adjuvants, the failure rate of primary retinal detachment (RD) repair secondary to proliferative vitreoretinopathy (PVR) has remained for the most part constant over the last few decades, up to 10% (Joeres et al. 2006; Sundar et al. 2018). It is the theory of the author that posterior vitreous detachment (PVD) with vitreoschisis can leave vitreous cortex remnants (VCR) over the retinal (mid)periphery (pVCR) and that these VCR promote the formation of PVR membranes which are pivotal in retinal redetachment. As such, visualization and removal of pVCR should improve surgical outcomes.

During spontaneous or surgical PVD, the inner lamellae of the posterior vitreous cortex can separate from the retina while the outermost lamellae remain attached to the retinal surface as VCR, in effect leading to vitreoschisis (Sebag 2008). These VCR can act like a scaffold for fibrocellular proliferation, while hyalocytes present in VCR may play a role in the development of an inflammatory response and membrane formation. It is postulated that pVCR through these mechanisms lead to the development of PVR detachments.

Previous studies focused on macular VCR (mVCR), which may give the impression that VCR only lead to macular pathology, especially in highly myopic and diabetic eyes (Sonoda et al. 2004; Sakamoto & Ishibashi 2009; Sebag et al. 2014). The author suggests that in fact VCR can be much more widespread across the retina and is a far more common finding than is presently detected. In order to visualize pVCR during vitrectomy, extensive targeted staining with triamcinolone is necessary. Given that triamcinolone is not routinely used for vitreous removal, the presence of pVCR is more than likely underestimated and more importantly missed, leading to an increased risk of PVR detachments.

A retrospective pilot study of two cohorts of consecutive patients who underwent vitrectomy for primary RD in 2016 (Cohort 1) and 2018 (Cohort 2) was performed to assess the prevalence of pVCR and its role in surgical failure. All vitrectomies were performed by a single surgeon with indentation and shaving of the vitreous base and the use of triamcinolone to enhance visualization of vitreous at the vitreous base and to attempt to detect pVCR. All patients had a follow‐up of at least 6 months.

In 2016 and 2018, respectively, initial PVR (≥C) was present and removed in 6/91 (7%) and 4/68 (6%) patients, pVCR were visualized in 26/91 (29%) and 30/68 (44%) patients, pVCR were removed in 16/26 (62%) and 21/30 (70%) patients with pVCR, and 3/26 (12%) and 7/30 (23%) patients with pVCR were high myopes (>6D). Retinal redetachment rates were 4/91 (4%) and 0/68 (0%), respectively.

In 2016, 3/26 (12%) patients with pVCR developed a redetachment compared to 1/65 (2%) patients without pVCR. In each of the three cases with pVCR and redetachment, pVCR were not completely removed during the first operation and a causative PVR membrane was identified during the second operation. No PVR had developed in the redetachment case without pVCR .

Differences between 2016 and 2018 could be attributed to more extensive and targeted staining (better detection) and more effective and safer removal of pVCR in 2018, using a new technique for pVCR removal, Vitreous Wiping (Van Overdam et al. 2018). The findings of this preliminary study support the theory that pVCR are more prevalent than previously thought (not only in highly myopic or diabetic eyes), that pVCR play a role in PVR development, and that visualization and removal of pVCR can improve the surgical outcome and reduce retinal redetachment rates.

**Figure 1:**
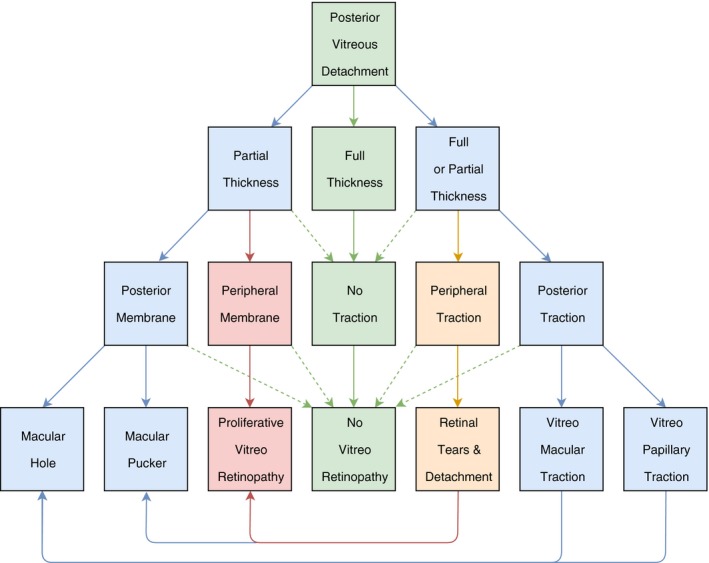
Updated, redesigned version of the schematic diagram of anomalous PVD (Sebag et al. 2014), including the ‘missing link’ (indicated in red). The original version showed that PVD can be full thickness (without VCR) or partial thickness (vitreoschisis with VCR). Full thickness PVD may lead to peripheral traction (which may result in a retinal tear and detachment), posterior traction (which may result in vitreomacular or vitreopapillary traction/adhesion and macular hole) or no traction (without vitreoretinopathy). Partial thickness PVD may lead to VCR over the macula (posterior membrane), which may result in a macular hole or macular pucker (depending on membrane thickness, presence of hyalocytes and vitreopapillary adhesion). In this updated version, the diagram is completed by adding that not only full, but also partial thickness PVD can lead to peripheral and posterior traction, but most importantly by adding the ‘missing link’: anomalous PVD with vitreoschisis can lead to VCR over the (mid)peripheral retina (peripheral membrane), which can act like a scaffold for fibrocellular proliferation, in which RPE cells from retinal tears and hyalocytes in VCR, together with previously identified PVR risk factors, conspire to form PVR membranes.

Certainly, larger prospective studies are required to further support and confirm these findings, but the author suggests that a ‘missing link’ in the pathophysiology of PVD and PVR has been revealed: VCR over the retinal (mid)periphery secondary to anomalous PVD with vitreoschisis. Figure 1 presents an updated and redesigned version of a previously published schematic diagram of anomalous PVD (Sebag et al. 2014), which includes this ‘missing link’.
